# Combined DSEK and Transconjunctival Pars Plana Vitrectomy

**DOI:** 10.1155/2016/9728035

**Published:** 2016-06-16

**Authors:** Mona Sane, Naazli Shaikh, Saad Shaikh

**Affiliations:** ^1^Orlando Health, Orlando, FL 32806, USA; ^2^Orlando VA Medical Center, Orlando, FL 32837, USA; ^3^Howard University College of Medicine, Washington, DC 20059, USA; ^4^University of Texas Medical Branch, Galveston, TX 77555, USA; ^5^University of Central Florida College of Medicine, Orlando, FL 32827, USA; ^6^University of South Florida College of Medicine, Tampa, FL 33620, USA; ^7^Florida State University College of Medicine, Tallahassee, FL 32304, USA

## Abstract

We report here three patients who underwent combined Descemet's stripping with endothelial keratoplasty and transconjunctival pars plana vitrectomy for bullous keratopathy and posterior segment pathology. A surgical technique and case histories are described. Anatomic and visual outcomes of combined Descemet's stripping with endothelial keratoplasty and vitrectomy were excellent. Our experience provides technical guidelines and limitations. The combined minimally invasive techniques allow for rapid anatomical recovery and return of function and visual acuity in a single sitting.

## 1. Introduction 

Par plana vitrectomy (PPV) has evolved into a minimally invasive, sutureless procedure with decreased operative times, reduced postoperative inflammation, and increased patient comfort. Likewise, Descemet's stripping with endothelial keratoplasty (DSEK), which selectively replaces diseased recipient endothelium with a healthy donor endothelial layer, is increasingly being used as an effective alternative to penetrating keratoplasty (PKP) for endothelial diseases. To our knowledge this is the first reported case series of simultaneous DSEK and pars plana vitrectomy in patients with advanced corneal decompensation and comorbid vitreoretinal pathology.

## 2. Surgical Technique

In all cases, vitrectomy was performed first by one surgeon (Saad Shaikh) followed by DSEK by another surgeon (Naazli Shaikh) under monitored anesthesia care with retrobulbar anesthetic injection (50 : 50 of 2% lidocaine and 0.75% bupivacaine). All patients underwent standard 23-gauge transconjunctival PPV and additional retinal procedures as necessary. In all cases, the corneal epithelium was debrided for better visualization. Thereafter, xenon light pipe illumination permitted sufficient visualization for the required vitreoretinal procedure. Additional lighting by chandelier systems was unnecessary. No patients underwent vitreous air-fluid exchange or gas injection. Once the retinal procedure was completed, the transconjunctival trocars were plugged and posterior infusion was maintained at low normal eye pressure. The DSEK donor lenticules were then prepared with a Moria artificial chamber (Moria, Antony, France), a Moria automated microkeratome (Moria, Antony, France) using a 300-micron depth, and a Moria Hanna Punch Block (Moria, Antony, France). The graft was punched at 8.5 mm in Cases 1 and 2 and 8.75 mm in Case 3. Following the usual steps of paracenteses, main corneal wound construction, scoring the endothelium with a reversed Sinskey hook (Bausch and Lomb Storz, San Dimas, California), and Descemet's membrane stripping and removal with a Moria reusable nonirrigating 90° spatula (Moria, Antony, France), the donor graft was introduced into the anterior chamber with a Busin Glide spatula (Moria, Antony, France). The corneal wound was closed using multiple interrupted 10-0 nylon sutures. Filtered air was then injected under the graft to unfold and center it. The graft was fixed peripherally using one to two, 10-0 prolene or nylon sutures. After a 10-minute delay to ensure adherence of the graft to the donor stromal bed, the scleral trocars were removed.

## 3. Case Series

### 3.1. Case 1

**Figure 1:**
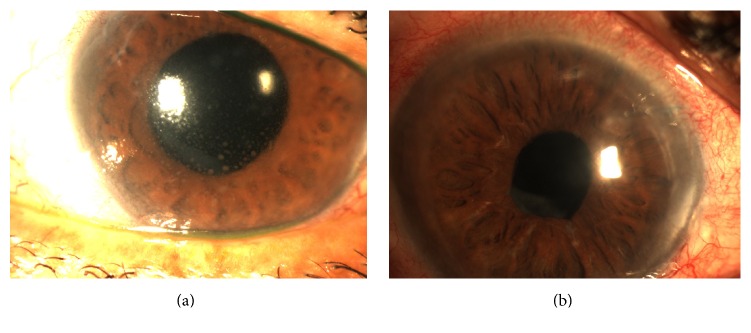
(a) Preoperative photo demonstrating corneal edema and keratic precipitates. (b) Postoperative photos demonstrating clear anterior chamber and cornea with stable DSEK graft and corneal stay suture.

A 61-year-old male with bilateral glaucoma and diabetic retinopathy presented with vision loss in his right eye. He had previously undergone focal laser therapy for diabetic maculopathy, cataract extraction with posterior chamber intraocular lens (PCIOL) implantation, and multiple intravitreal triamcinolone injections for chronic granulomatous uveitis of unknown etiology in the same eye. Best-corrected visual acuity was 8/200. Anterior segment examination demonstrated bullous keratopathy, a thickened cornea, and granulomatous iridocylitis (Figure 1(a)). Fundus examination revealed vitritis, multiple laser scars in the macula, intraretinal hemorrhages, and glaucomatous cupping of the disc with an intraocular pressure of 30 mmHg. Optical coherence tomography demonstrated macular edema. An uncomplicated diagnostic PPV with panretinal photocoagulation and pars plana endocyclophotoablation for glaucoma was performed followed by DSEK in a combined procedure. Although vitreous samples were negative, the host corneal tissue was positive for cytomegalovirus by polymerase chain reaction testing. The patient was subsequently placed on oral ganciclovir therapy. At six weeks, his visual acuity had improved to 20/400 with a clear and well-centered graft (Figure 1(b)). IOP was 17 mmHg and the anterior segment was free of cell and keratic precipitates. At 30 months' final follow-up both the cornea and vitreous cavity remained clear and best-corrected vision had improved to 20/100. Fluorescein angiography confirmed the presence of macular ischemia limiting final visual acuity.

### 3.2. Case 2

**Figure 2:**
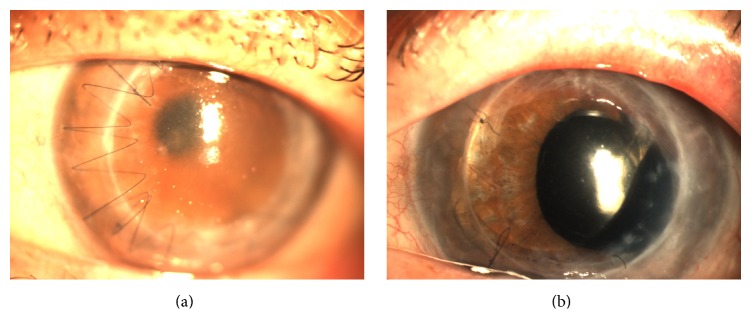
(a) Preoperative photo demonstrating failed PKP with corneal edema. (b) Postoperative photos demonstrating ACIOL with clear cornea and stable DSEK graft.

An 80-year-old male with Fuch's endothelial dystrophy, history of complicated cataract extraction, chronic cystoid macular edema, and two previously failed PKPs presented with graft rejection and a best-corrected visual acuity of 20/400 OS (Figure 2(a)). The patient underwent an attempted DSEK procedure by one of the authors (Naazli Shaikh) under the preexisting PKP graft which was aborted when the donor lenticule could not be unfolded in the anterior segment due to vitreous prolapse and cortical material presenting in the anterior chamber around a subluxed PCIOL. The patient then underwent a combined DSEK, pars plana vitrectomy, and intraocular lens (IOL) exchange one week later. A standard core and peripheral vitrectomy for retained cortical fragments and vitreous fibrosis was performed. Vitreous adhesions to the subluxed lens were released and the PCIOL was removed through a temporal clear corneal wound. An anterior chamber intraocular lens (MTA4U0, Alcon Surgical, Fort Worth, Texas) was placed into the anterior chamber and thereafter DSEK was performed. The postoperative period was uneventful, and at 31 months' final follow-up the graft remained clear (Figure 2(b)) with best-corrected vision of 20/70, limited by foveal pigmentary changes and atrophy.

### 3.3. Case 3

**Figure 3:**
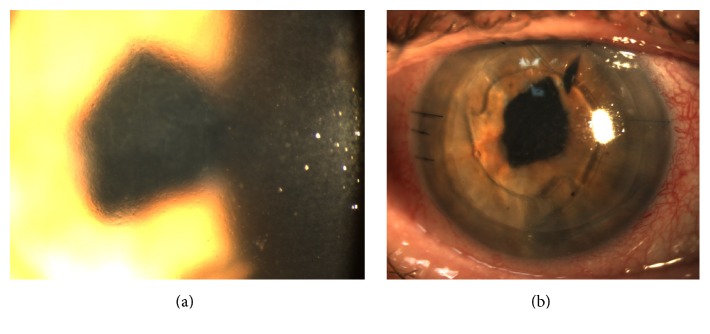
(a) Preoperative photo (above) demonstrating corneal edema. (b) Postoperative photos with ACIOL, clear cornea, and stable DSEK graft with corneal stay suture.

A 77-year-old male with history of complicated cataract surgery in his right eye presented with decreased vision from pseudophakic bullous keratopathy (Figure 3(a)). Visual acuity was 20/200. Anterior segment examination demonstrated an anterior chamber intraocular lens (ACIOL), a large superior sector iridectomy, and vitreous strands enveloping the ACIOL. Anterior hyaloid proliferation and fibrous membranes were noted behind residual lens and capsule remnants. A pars plana vitrectomy to remove cortical remnants, anterior hyaloid membranes, and vitreolenticular adhesions was combined with a DSEK procedure. On postoperative day one, the air bubble had moved to the posterior segment and the superior edge of the graft showed some separation from the host but remained centered against the host cornea. Filtered air was injected into the anterior chamber at the slit lamp, but the air bubble traveled immediately into the posterior segment again through the preexisting iridectomy. The patient was brought to the operating room. A small paracentesis site was made superiorly, and a McCannel stitch (10-0 prolene) was used to close the peripheral component of the superior sector iridectomy. Filtered air was injected into the anterior chamber, and the air bubble and graft remained stable in the postoperative period. At 16 months' final follow-up, the graft remained clear and centered with best-corrected visual acuity of 20/100 (Figure 3(b)).

## 4. Discussion

Patients in this series required both vitrectomy for diagnostic or therapeutic purposes and DSEK surgery for corneal decompensation. All patients were successfully treated using a combined surgical technique. One patient required return to operating room for repair of an iridectomy that allowed posterior communication of the anterior chamber bubble. No intraoperative or postoperative complications were noted. All patients achieved excellent anatomic outcomes and visual improvement consistent with preexisting retinal pathology. Our experience here provides technical guidelines for successful combined DSEK and vitreoretinal surgery.

Consideration of retinal surgical indications is critical for selecting combined patients. Combined surgery is not suitable for retinal cases requiring postoperative air tamponade (i.e., macular holes) for several reasons. DSEK requires face up positioning to secure attachment of the corneal graft while retinal cases typically require face down or to the side positioning. Posterior pressure on the lens/iris complex from air left in the vitreous cavity at the end of the case may cause iridocorneal touch, especially in aphakic eyes, and this could displace the corneal graft or prevent its adhesion. Furthermore, retinal operative viewing may be limited by corneal pathology. The combined approach should only be considered if the corneal clarity is sufficient for posterior segment visualization by the retinal surgeon. Some degree of corneal haze can be overcome by epithelial debridement and/or using higher illumination settings, xenon lighting, or chandelier systems. Combined surgery may not be optimal for cases with silicone oil. The effect of silicone oil on DSEK grafts has not been adequately studied. Oil emulsification and migration between the host stroma and DSEK graft may affect graft adhesion and survival. It should also be noted that aphakic eyes or eyes with anterior chamber lenses may require multiple repeat air injections in the anterior chamber to secure the corneal graft due to posterior migration of the bubble, and patients with large peripheral iridotomies or postsurgical iris defects that could allow passage of the anterior chamber gas bubble into the vitreous cavity will require additional intraoperative iris reconstruction.

Graft dislocation is a potential complication of DSEK surgery. Meticulous surgical technique can help to mitigate this risk [1, 2]. Graft dislocation into the posterior segment is a direct risk factor for proliferative vitreoretinopathy induced retinal detachment, typically in aphakic, ACIOL, or sutured IOL eyes [3]. Because of this, all patients in this series underwent donor graft fixation sutures in order to prevent such complications. The procedure, previously described, is simple and reduces primary graft failure rates [4, 5].

All procedures were completed in an outpatient setting using only sedation and retrobulbar anesthetic. Combining DSEK with vitrectomy in the appropriate clinical situation facilitates faster visual recovery, avoids separate surgeries and repeated anesthesia, decreases costs, and is ultimately more comfortable for the patient. Case selection and consideration of surgical pathology are critical in selecting patients for combined DSEK and vitrectomy procedures. Notwithstanding, our experience here has demonstrated that such procedures can have successful anatomic and visual outcomes.
